# Multiorbital Two-Band Landau–Fermi Liquidness of 1*T*‑Ti(Se,Te)_2_ van der Waals Crystals

**DOI:** 10.1021/acs.inorgchem.5c04404

**Published:** 2026-02-03

**Authors:** Luis Craco, Bo Hou, Stefano Leoni

**Affiliations:** † Institute of Physics, 67826 Federal University of Mato Grosso, 78060-900 Cuiabá, Mato Grosso, Brazil; ‡ School of Physics and Astronomy, 385187 Cardiff University, Cardiff CF24 3AA, U.K.; § School of Chemistry, 385187 Cardiff University, Cardiff CF10 3AT, U.K.

## Abstract

Normal-state Landau–Fermi-liquid (LFL) behavior is widely regarded as a prerequisite for low-temperature superconductivity in 1*T*-Ti*X*_2_ (*X* = Se, Te) van der Waals (vdW) crystals. Clarifying this role requires a microscopic description of how local electron correlations and Ti–chalcogen covalence cooperate to shape the low-energy electronic structure in the noncharge-density-wave (non-CDW) regime. In the present work, we employ density functional theory combined with dynamical mean-field theory (DFT + DMFT) to investigate an extended multiorbital (MO) two-band Hubbard model specifically constructed for these transition-metal dichalcogenides. The calculations reveal an emergent LFL metal stabilized by dynamical intra- and interorbital correlations in the Ti-based manifold, while the chalcogen 4p/5p states remain comparatively rigid against changes in interaction strength. This orbital-selective reconstruction leads to a strongly anisotropic renormalization of the Ti-3d sector, which we identify as a key ingredient for the superconducting phase diagram of 1*T*-Ti*X*_2_. Beyond demonstrating the capability of DFT + DMFT to capture such MO correlation effects, our results show that proximity to a correlated LFL state naturally accounts for the distinct low-temperature transport responses of the Se and Te compounds, where modest variations in interaction-to-bandwidth ratio and orbital occupancy drive markedly different sensitivities to external tuning parameters such as pressure, doping, or gating.

## Graphical abstract

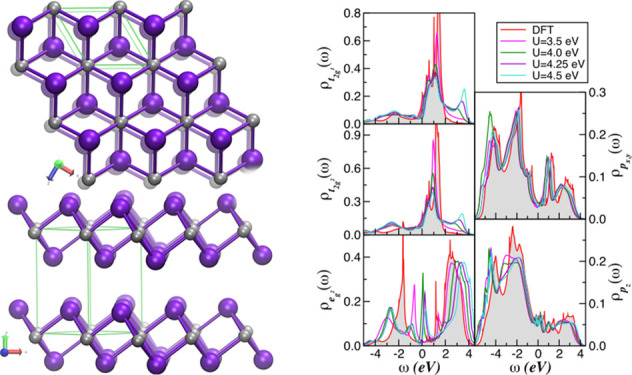

## Introduction

The emergence of unconventional quantum phases from correlated electrons is one of the central themes in contemporary condensed-matter physics. A recurring motif in such systems is the delicate balance between lattice geometry, electronic bandwidth, and Coulomb interactions, which collectively control whether the low-energy charge carriers behave as long-lived quasiparticles or as incoherent excitations. In the simplest scenario of a paramagnetic metal, this balance can give rise to a Landau–Fermi-liquid (LFL) state,^1,2^ typically realized when the local interaction strength *U* is comparable to the bare one-particle bandwidth *W*, i.e., when the effective ratio *U*/*W* approaches unity.

A large body of experimental and theoretical work has demonstrated that the qualitative influence of electron–electron interactions is governed by the ratio *U*/*W*. For small *U*/*W*, the transport and spectral properties are primarily encoded in the one-electron hopping and, if present, electron–phonon coupling.^3^ As *U*/*W* increases toward order one, correlation effects become increasingly prominent, and in the large-*U*/*W* limit, double occupancy is strongly suppressed, eventually driving a Mott insulating state at half filling.^4,5^ In this regime, modest external tuning—for example pressure, carrier doping or disorder—can trigger dramatic changes in the electronic ground state and stabilize competing phases.^6^ Within this broader context, the 1*T* polymorph of TiTe_2_ stands out as a reference correlated LFL metal,^7–10^ where charge-density-wave (CDW) order and superconductivity can coexist at low temperature,^11,12^ similar to the related 1*T*-TiSe_2_ compound.^13,14^

Layered transition-metal dichalcogenides (TMDs) of composition *MX*_2_ (M = Ti, Zr, Hf, V, Nb, Ta, Mo, W, Re; *X* = S, Se, Te) provide an ideal platform to explore such correlation phenomena in quasi-two-dimensional crystals. Each structural unit consists of a hexagonal transition-metal layer sandwiched between two chalcogen layers, forming an *X*–*M*–*X* triple layer. These units are stacked along the *c* direction and held together by relatively weak vdW forces, while the in-plane *M*–*X* bonds are strongly covalent and directional. Depending on the particular *M* and *X*, the local coordination of the transition-metal ion is either trigonal prismatic or octahedral with six surrounding chalcogens.^15^ In several TMDs, superconductivity can be tuned or induced by inserting ions or molecules into the vdW gaps,^16–18^ by applying hydrostatic or chemical pressure,^19,20^ or via electrostatic gating in thin flakes.^21^

When charted as a function of external control parameters such as doping or pressure, the phase diagrams of several TMDs bear a striking resemblance to those of high-*T*_c_ cuprates, iron-based superconductors, and heavy-fermion compounds: superconducting domes often appear in the vicinity of competing CDW or other symmetry-broken states, suggesting a nontrivial involvement of dynamical electronic correlations.^22^ For the 1*T*-Ti*X*_2_ (*X* = Se, Te) family, this picture implies that a correlated LFL metal serves as the parent state from which CDW order and superconductivity emerge. However, a microscopic understanding of how this LFL state is stabilized, and how it differs between TiSe_2_ and TiTe_2_ is still lacking. The present work addresses this issue by explicitly disentangling the role of multiband and multiorbital correlations in the normal, non-CDW phase of 1*T*-Ti*X*_2_.

Titanium-based dichalcogenides Ti*X*_2_ (*X* = S, Se, Te) have attracted renewed attention due to their rich combination of structural, electronic, and optical properties.^23–30^ Beyond their role as model systems for correlated-electron physics, these compounds are promising building blocks for applications ranging from lithium-ion batteries^31^ and resistive random-access memories^32,33^ in neuromorphic architectures,^34^ to solid-state cooling concepts based on thermoelectric and caloric effects.^35^ In this broader application landscape, understanding how correlations renormalize their normal-state transport response has become increasingly relevant.

As in other vdW crystals, the stacking of *X*–Ti–*X* units leads to a pronounced anisotropy between strong in-plane bonding and weak out-of-plane coupling,^24^ and hence to quasi-two-dimensional electronic structures. For 1*T*-Ti*X*_2_, the high-temperature phase and the microscopic origin of the CDW instability remain under active debate,^29,36^ while transport data reveal a number of unconventional features.^23,37,38^ In a conventional CDW material, the opening of a gap at Fermi energy *E*_F_ typically produces a monotonic increase in resistivity upon cooling through the transition. By contrast, in 1*T*-TiSe_2_, the resistivity first rises below ∼205 K and then drops steeply around 165 K, a nonmonotonic behavior that has prompted several competing interpretations ranging from Fermi-surface reconstructions^39^ to temperature-dependent carrier densities and correlation-driven renormalizations of the quasiparticle mass.^40,41^

An additional hallmark of 1*T*-TiSe_2_ and 1*T*-TiTe_2_ is the observation of a nearly quadratic LFL-like resistivity, ρ­(*T*) ∝ *T* ^2^,^7,9,10,13,14^ either within the CDW phase or below a material- and sample-dependent inflection point, reminiscent of the behavior reported for Sr_2_RuO_4_.^42^ Optical spectroscopy further corroborates this picture by revealing a strong enhancement of the Drude weight in the CDW phase,^43^ consistent with coherent quasiparticles whose dynamics is strongly influenced by self-energy corrections,^44^ similarly to what has been established in 2*H*-TaSe_2_. These observations point toward a subtle interplay between multiband, multiorbital correlations, and ordering tendencies^22,45,46^ and motivate a detailed theoretical analysis of the normal-state LFL metal in 1*T*-Ti*X*_2_.

Motivated by this, we formulate and analyze an extended two-band Hubbard model, closely related in spirit to that previously introduced for 2*H*-TaSe_2_,^22^ but here explicitly constructed from ab initio electronic-structure data for 1*T*-TiSe_2_ and 1*T*-TiTe_2_. By solving this model within a multiorbital DFT + DMFT framework, we uncover how dynamical correlations reshape the Ti-3d and chalcogen-*p* manifolds in the non-CDW metallic phase and thereby stabilize an orbital-selective LFL state. The resulting picture provides a microscopic basis for understanding the correlated normal state from which CDW order and superconductivity develop in this family of vdW materials.

## Computational Methods

The Ti*X*_2_ (*X* = Se, Te) compounds crystallize in the hexagonal 1*T* structure (space group *P*3-*m*1, No. 164). As indicated in Figure 1, Ti atoms occupy the Wyckoff site 1*c* at the origin, while the two chalcogen atoms reside on the 2*d* positions at ± (1 3, 2 3, **z**). To obtain realistic low-energy Hamiltonians for these systems at ambient pressure, we first carry out ab initio density functional theory (DFT) calculations using the experimental lattice parameters *a* and *c* for both 1*T*-TiSe_2_ and 1*T*-TiTe_2_, representative members of the group-IVB TMD family.^10^ The electronic structure is computed within the PBE generalized-gradient approximation employing Vanderbilt ultrasoft pseudopotentials, as implemented in the pw.x code (v7.2) of the Quantum Espresso package.^47^ The plane-wave basis is truncated at kinetic energy cutoffs of 50 Ry for the wave functions and 280 Ry for the charge density. Self-consistent calculations are performed on a 14 × 14 × 8**k**-mesh. For 1*T*-TiSe_2_, Se occupies the 2*d* site at (1/3, 2/3, 0.7329), while for 1*T*-TiTe_2_ the Te coordinate is (1/3, 2/3, 0.737).

**Figure 1:**
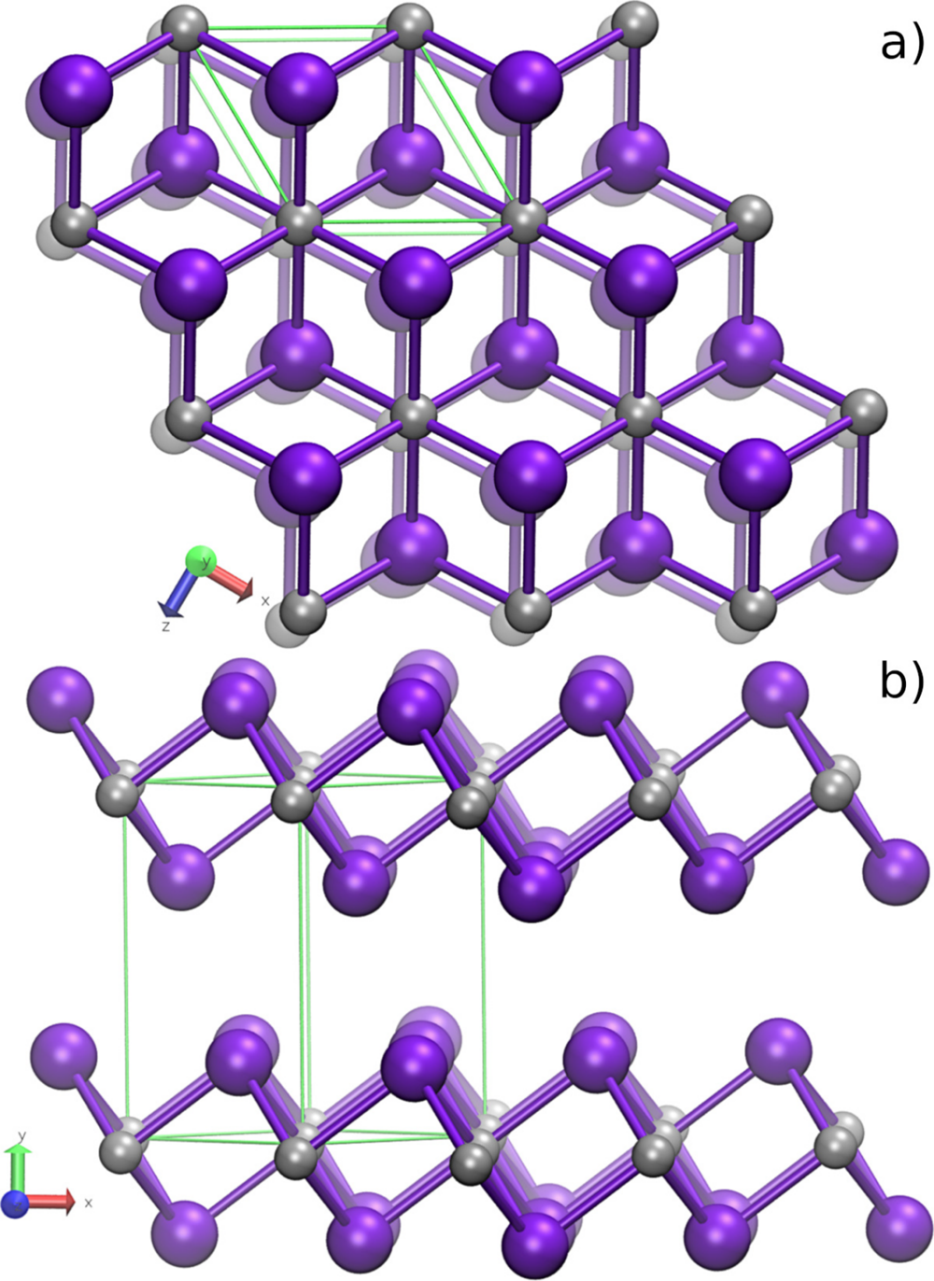
1*T*-Ti*X*_2_ layered structure. (a) View perpendicular to the plane of the layers, emphasizing the eclipsed arrangement of the layers as well as the local Ti coordination. (b) *X*-Ti-*X* layers are stacked vertically, with Ti (silver spheres) octahedrally coordinated by 6 *X* atoms (*X* = Se or Te, purple spheres). Ti atoms are sandwiched between two Se/Te layers. The hexagonal unit cell is drawn in green.

To construct a tight-binding representation suitable for many-body treatments, we generate maximally localized Wannier functions (MLWFs) using the Wannier90 code^48^ (v3.1). As initial projections, we choose five Ti 3d-like orbitals per primitive cell and include the Se 4p or Te 5p valence states, resulting in a total of 11 MLWFs. The Wannierization is performed within an energy window from 9.40 to 12.8 eV, which is chosen to ensure localized, atomic-like Ti-centered Wannier functions with the appropriate site symmetry, while faithfully reproducing the chalcogen-derived states. Spread minimization yields real-valued MLWFs that accurately interpolate the DFT bands in the energy window relevant for the low-energy physics.

The comparison between the interpolated Wannier bands and the original DFT dispersions is displayed in Figure 2 for the 11-band model together with the corresponding total DFT density of states (DOS) for 1*T*-TiSe_2_ and 1*T*-TiTe_2_ in their normal, non-CDW phase. The agreement with previous band-structure studies^24,10^ confirms that the Wannier Hamiltonians capture the relevant low-energy physics of both compounds.

**Figure 2:**
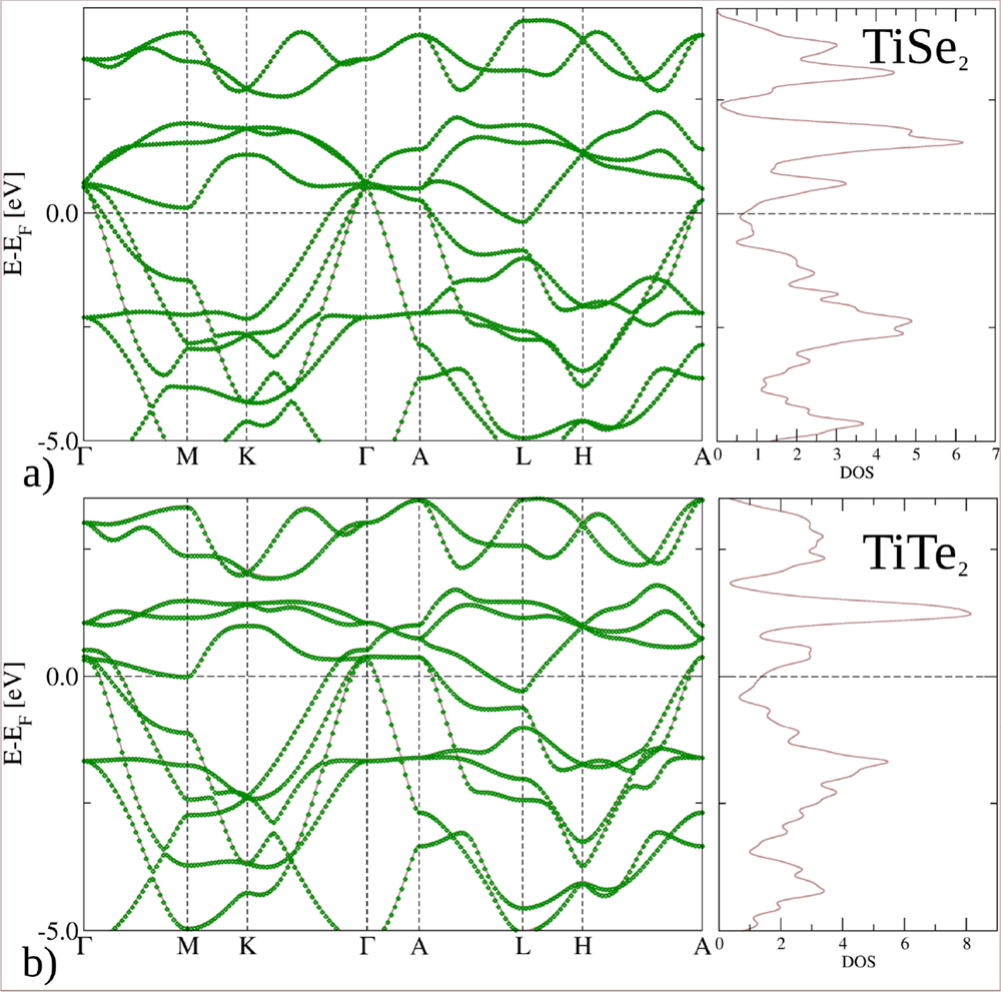
*k*-Dependent band structures and total DOS of Ti*X*_2_ (*X* = Se,Te) van der Waals crystals in the normal, non-CDW ordered state. The Wannier models for (a) TiSe_2_ and (b) TiTe_2_ are shown as green diamonds, superimposed onto Bloch states (brown lines). The number of *k*-points for the Wannier model was downsampled for clarity.

In Figure 3, we show the atom- and orbital-resolved DFT DOS over the energy range most relevant to correlation effects. In agreement with earlier work,^26,27,30^ both compounds display a finite DOS at the Fermi level, consistent with metallic behavior. The projected DOS reveals substantial contributions from Ti-3d and chalcogen 4p/5p states at *E*_F_ and highlights several van Hove-like structures originating from relatively flat bands in the underlying dispersion (not shown).^26^ A Bader charge analysis, in line with ref 27, indicates that each Ti atom transfers about 0.65 (0.8) electrons to each Se (Te) ion, corresponding to total charges of approximately 1.3 (1.6) electrons donated to the two chalcogens, underscoring the mixed ionic–covalent character of the bonding. Comparing the Se and Te cases, we find a modest reduction of the one-particle bandwidth *W* in the Te compound and a more pronounced valence peak in the Ti e_g^2^_ channel in 1*T*-TiTe_2_, features that will play an important role in the correlation-driven spectral reconstruction discussed below.

**Figure 3:**
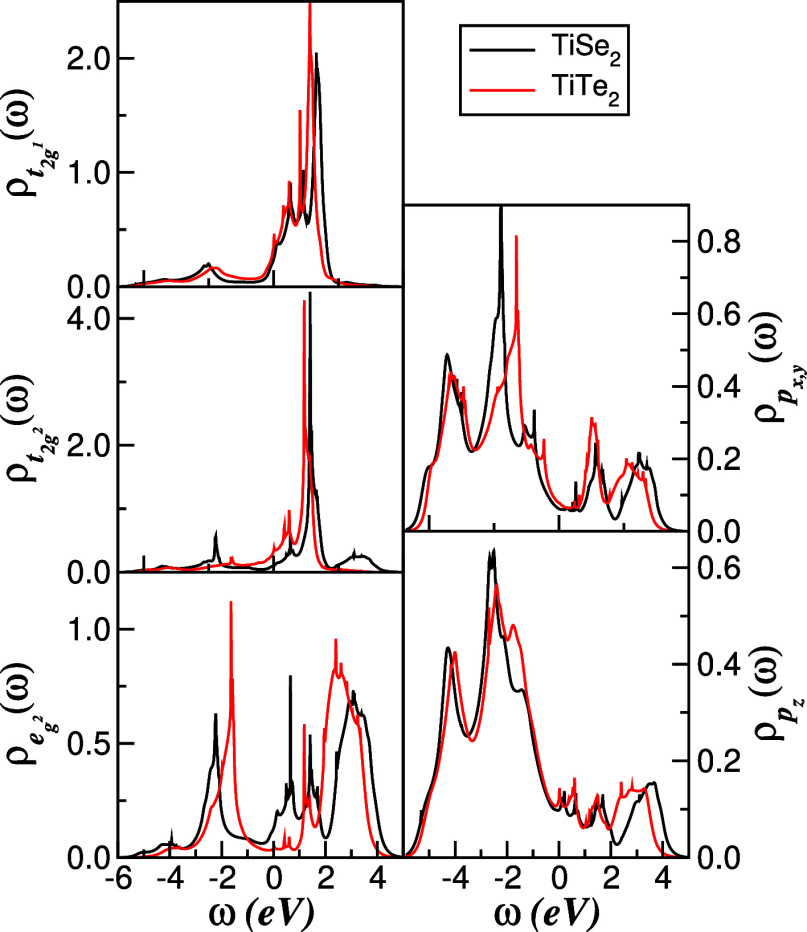
Projected DFT DOS of Ti*X*_2_ (*X* = Se,Te) van der Waals crystals in the normal, non-CDW ordered state. Note the pronounced particle–hole asymmetry and the presence of van Hove-like peaks at different energies. The Te compound exhibits an overall bandwidth slightly narrower than that of the Se analogue.

The strong sensitivity of these DOS profiles to local interactions makes them an ideal starting point for dynamic mean-field analysis. In the following, we therefore combine the Wannier Hamiltonians with an extended multiorbital Hubbard interaction and treat the resulting problem within DFT + DMFT.^49^ This allows us to quantify how electron–electron correlations, multiband couplings, and orbital selectivity reshape the spectral functions beyond the DFT level.

The one-electron part of the effective Hamiltonian for each atomic channel α [α = (1, 2)] can be written as

$$H_{\alpha }^{0}=\underset{k,a,\sigma }{\sum }\epsilon_{\alpha ,a}\left(k\right)c_{\alpha ,k,a,\sigma }^{†}c_{\alpha ,k,a,\sigma }$$

where *a* labels the diagonalized (in the orbital basis) Ti-3d and *X*-p Wannier orbitals. Within this two-band description of 1*T*-Ti*X*_2_, *c*_α,*i*,*a*,σ_^†^ (*c*_α,*i*,*a*,σ_) creates (annihilates) an electron of spin σ in orbital *a* of atomic channel α at site *i*, *n*_α,*i*,*a*,σ_ = *c*_α,*i*,*a*,σ_^†^ *c*_α,*i*,*a*,σ_, and ϵ_α,*a*_(**k**) encodes the band dispersions obtained from Wannier interpolation.

Local two-particle interactions on each atomic channel are described by

$$H_{\alpha }^{\mathrm{int}}=U\sum_{i,a}n_{\alpha ,i,a,↑}n_{\alpha ,i,a,↓}+U^{\prime }\sum_{i,a\neq b}n_{\alpha ,i,a}n_{\alpha ,i,b}-J_{H}\sum_{i,a\neq b}S_{\alpha ,i,a}\cdot S_{\alpha ,i,b}$$

where *U* and *U′* denote the intra- and interorbital Hubbard repulsions and *J*_*H*_ = 0.7 eV is the Hund’s exchange coupling, with the usual relation *U′* = *U* – 2*J*_*H*_. The full interacting Hamiltonian for 1*T*-Ti*X*_2_ then reads

$$H=\sum_{\alpha }(H_{\alpha }^{0}+H_{\alpha }^{\mathrm{int}})$$

to which we add an intersite Ti–*X* interaction

$$H_{\mathrm{pd}}=\frac{U_{\mathrm{pd}}}{2}\sum_{<ij>a,\sigma ,\overset{®}{\sigma }}n_{i,a,\sigma }n_{j,a,{\overset{®}{\sigma }}^{\prime }}$$

where <*ij*> denotes nearest-neighbor Ti–*X* bonds and *U*_pd_ is the corresponding interband Coulomb repulsion. In the present work, we set *U*_pd_ = 0.5*U*, in line with previous studies of extended Hubbard models.^50^ Following standard practice for high coordination,^51–53^ we treat *H*_pd_ at the Hartree level, which becomes exact in the large-dimensionality limit.^2^ The resulting extended two-band Hamiltonian *H*– = *H* + *H*_pd_ thus captures both local multiorbital correlations and an effective orbital-dependent shift due to intersite Coulomb interactions.

Within DMFT,^2,49^ the lattice problem defined by *H*– is mapped onto a self-consistent multiorbital Anderson impurity model. The many-particle Green’s functions

$$G_{\alpha ,a,\sigma }\left(\omega ,k\right)=\frac{1}{\left[\xi_{\alpha ,a,\sigma }\left(\omega \right)-\epsilon_{\alpha ,a}\left(k\right)\right]}$$

with ξ_α,*a*,σ_(ω) ≡ ω + *i*η – Σ_α,*a*,σ_(ω + *i*η) are obtained from the impurity self-energies Σ_α,*a*,σ_(ω) and iterated until the local impurity Green’s function coincides with the local lattice Green’s function. To solve the multiorbital DMFT equations, we employ a multiorbital iterated-perturbation-theory (MO-IPT) impurity solver,^54^ which provides numerically efficient access to the self-energy over a broad frequency range and has proven reliable for systems with multiple *d* and *p* orbitals.^55^ Given the complexity of the Ti-3d and *X*-p manifolds in 1*T*-Ti*X*_2_, this approach offers a practical compromise between accuracy and computational cost while retaining the essential dynamical correlation physics.

## Results and Discussion

Previous theoretical efforts to describe many-body effects in 1*T*-Ti*X*_2_ (*X* = Se, Te) have employed a variety of beyond-DFT approaches, including ab initio *GW*,^56^ quasiparticle self-consistent GW (QSGW),^57^ quasi-self-consistent *G*_0_ *W*_0_,^58^ and GGA + *U*.^26^ In the latter work, a moderate Hubbard interaction *U* = 3.0 eV applied to the Ti-3d manifold was sufficient to improve the agreement between calculated and measured band dispersions in the 0–2 eV binding-energy window. Complementary insight into the role of interband interactions has been obtained from variational Monte Carlo simulations of a two-band Hubbard model on a triangular lattice,^50^ highlighting how local and nonlocal Coulomb terms conspire to reshape the correlated band structure. Building on these developments, our DFT + DMFT treatment of the Wannier-derived two-band model allows us to analyze the correlated spectral functions and self-energies directly in the orbital-resolved basis relevant for Ti*X*_2_.

In Figures 4 and 5, we present, respectively, the evolution of the orbital- and channel-resolved DOS and the corresponding imaginary part of the self-energy for 1*T*-TiSe_2_ as the Hubbard interaction *U* is varied between 4.5 and 5.5 eV. In this parameter range, where *U*/*W* ≈ 0.5, the Se-derived 4p states are only weakly affected by the local correlations due to their almost filled character and strong polarization in the noninteracting DOS, and thus effectively act as a broad, nearly uncorrelated electron reservoir. In the Ti-3d sector, by contrast, the various t_2g_ and e_g^2^_ orbitals respond very differently to interactions. Although the t_2g_ orbitals have similar occupancies overall, the t_2g^1^_ orbital exhibits a much stronger redistribution of spectral weight: as *U* increases, spectral weight near *E*_F_ is progressively transferred into an upper Hubbard band (UHB), signaling the onset of incoherent high-energy excitations. The e_g^2^_ channel shows an even more pronounced reconstruction due to its enhanced valence-band occupancy, which is itself promoted by sizable Ti–*X* hybridization.^59^ The bonding-like feature at around −2.3 eV in the e_g^2^_ DOS splits into a lower Hubbard band (LHB) and a shoulder that shifts toward *E*_F_ with increasing *U*. Because the low-energy self-energy imaginary parts vanish quadratically in frequency (Figure 5), this shoulder evolves into a narrow Kondo-like quasiparticle resonance,^2^ characteristic of a correlated Fermi liquid governed by intertwined spin, charge, and orbital fluctuations.

**Figure 4:**
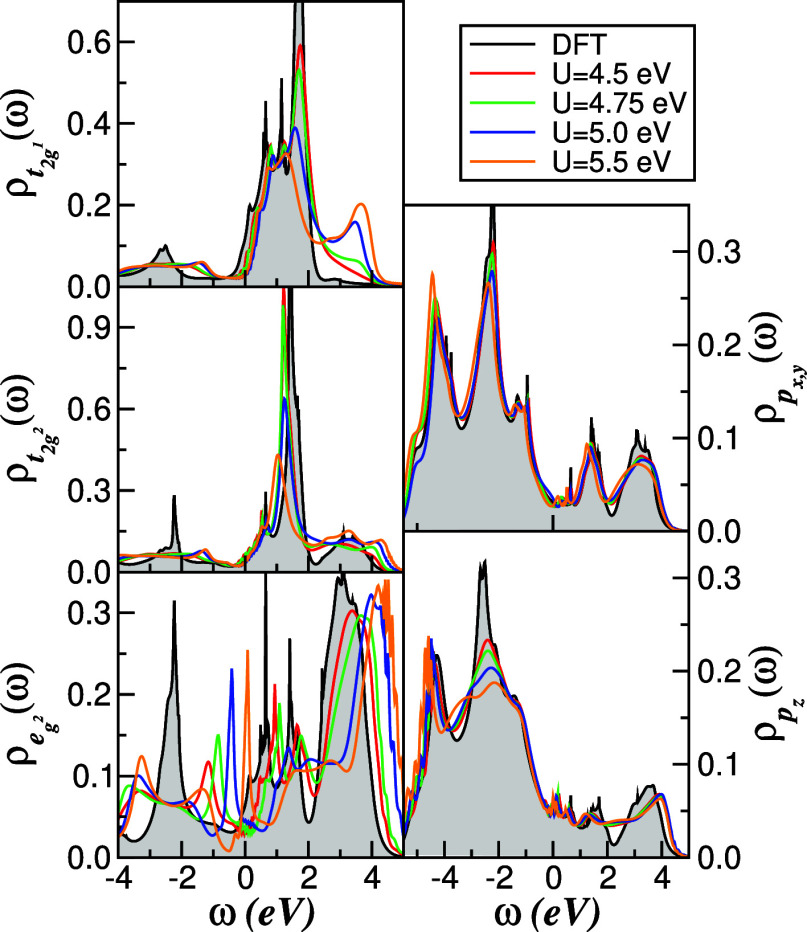
*U*-dependence of the orbital- and channel-resolved DOS within the non-CDW phase of 1*T*-TiSe_2_. Increasing *U* enhances Hubbard sidebands in the Ti-derived channel and drives a pronounced reconstruction of the e_g^2^_ spectral weight from low to high energies via dynamic spectral-weight transfer. The Se-derived DOS is only weakly reshaped, indicating that it acts as a nearly free-electron reservoir in the correlated metal.

**Figure 5:**
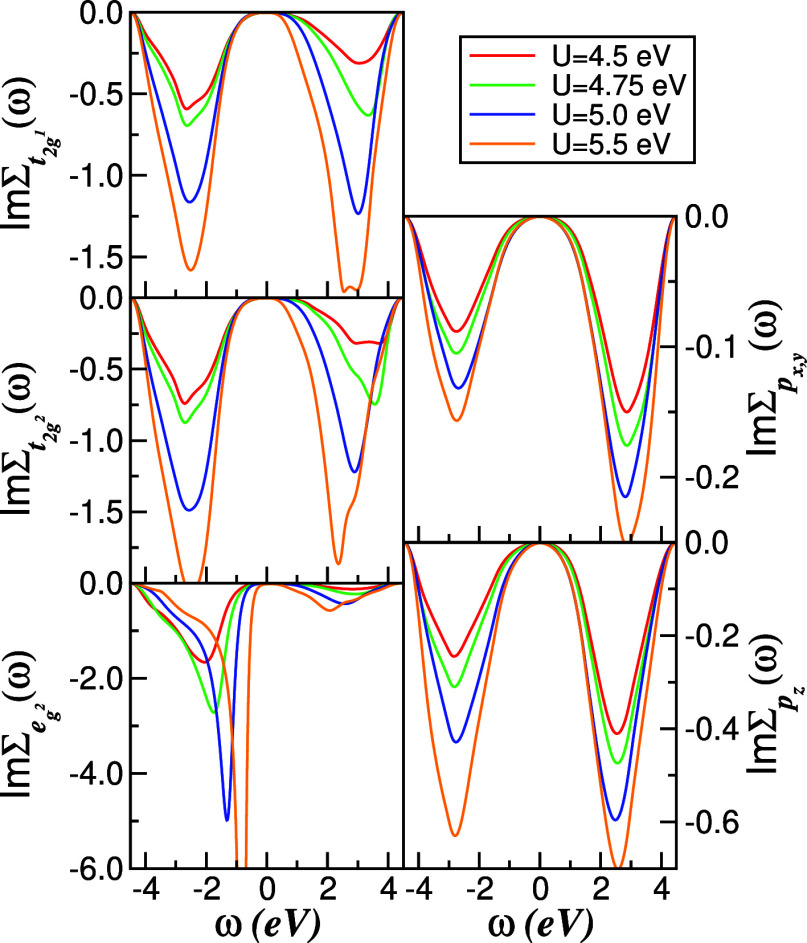
Energy dependence of the orbital- and channel-resolved self-energy imaginary parts for the two-band model of 1*T*-Ti*X*_2_, showing the ω^2^ behavior of Im Σ_α_(ω) close to the Fermi level, characteristic of a Landau–Fermi-liquid metal.^2^ Note also the pronounced particle–hole asymmetry and the stronger correlation fingerprints in $\Sigma_{e_{g^{2}}}(\omega )$ with increasing *U*.

Taken together, the spectral functions and self-energies in Figures 4 and 5 display the characteristic fingerprints of a multiorbital Fermi liquid within DMFT.^2^ In particular, the e_g^2^_ orbital develops well-formed Hubbard sidebands at high energies that coexist with a coherent resonance at *E*_F_, whereas the t_2g_ states remain comparatively less reconstructed but still exhibit bandwidth narrowing due to correlations. The multiorbital character is further reflected in the approximate pinning^2^ of the interacting spectral function at *E*_F_ in the t_2g_ sector, despite the strong particle–hole asymmetry of the underlying one-particle DOS. This orbital-selective behavior foreshadows the differences that will emerge between the Se and Te systems when the bandwidth is reduced.

To explore the impact of bandwidth reduction and chalcogen substitution, Figure 6 shows the orbital- and atom-resolved DFT + DMFT DOS for 1*T*-TiTe_2_ for a set of representative *U* values. Similar to the Se case, dynamical correlations induce a transfer of spectral weight from low to high energies and enhance the Hubbard satellites as *U* is increased. However, owing to the reduced bare bandwidth of the Te compound (Figure 3), the effective ratio *U*/*W* is larger, and correlation effects are correspondingly more pronounced. This manifests in more substantial orbital- and channel-selective spectral rearrangements: in particular, the van Hove singularity centered around 1.6 eV below *E*_F_ in the Ti e_g^2^_ channel is strongly suppressed and shifted as spectral weight is redistributed over a wider energy range. The right-hand panels of Figure 6 show that the Te-5p states, being almost fully occupied, are only weakly renormalized and display minimal correlation-induced features above *E*_F_. This behavior reflects the reduced effectiveness of Hubbard interactions away from half-filling^60^ and underlines the role of Ti-3d orbitals as the primary locus of strong correlations.

**Figure 6:**
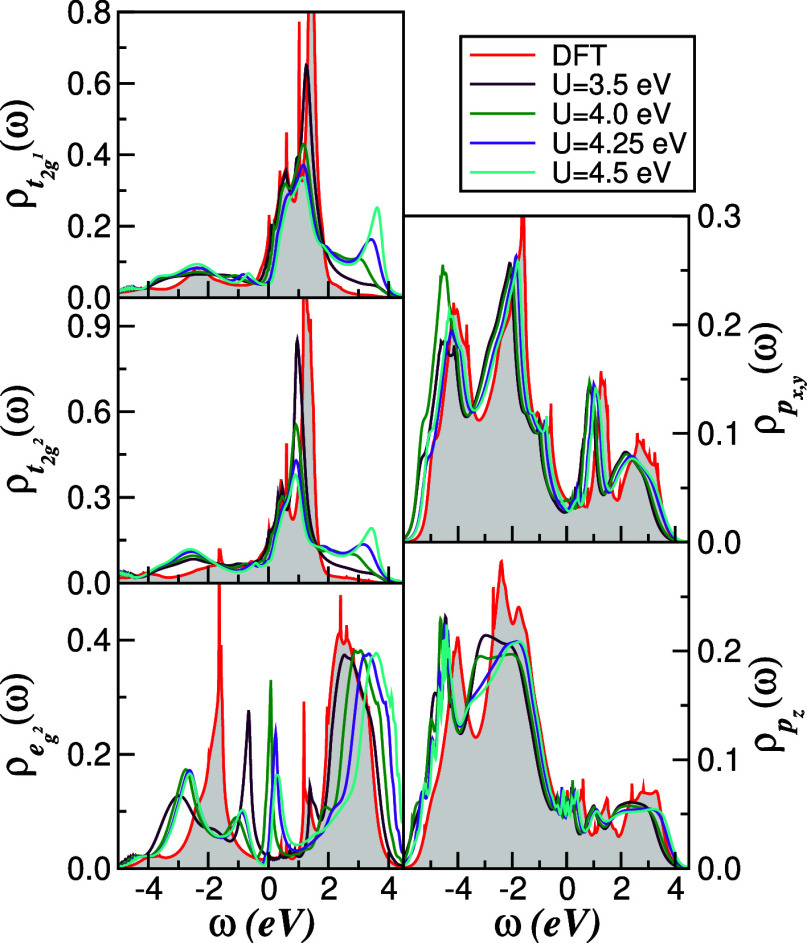
Evolution of the orbital- and channel-resolved DFT +DMFT DOS within the normal, non-CDW ordered state of 1*T*-TiTe_2_. The van Hove singularity in the Ti e_g^2^_ channel is strongly renormalized with increasing *U*, reflecting enhanced correlation effects in the Te compound.

Additional insight into the low-energy coherence of 1*T*-TiTe_2_ is provided by the frequency dependence of the Ti-3d and Te-5*p* self-energies shown in Figure 7. As in the Se compound (Figure 5), the imaginary part Im Σ_α,*a*_(ω) for *a* = t_2g^1,2^_, *p*_*x*,*y*,*z*_ exhibits a clear ω^2^ dependence at small frequencies, signaling the presence of LFL quasiparticles across all channels.^2^ The corresponding real parts (not shown) vary linearly with ω near *E*_F_ and yield sizable mass enhancements, particularly in the more strongly correlated e_g^2^_ orbital. Combining these observations with earlier DMFT studies of multiorbital metals,^2,54^ we conclude that 1*T*-TiTe_2_ at ambient pressure realizes a correlated Fermi liquid with enhanced orbital selectivity relative to 1*T*-TiSe_2_. Increasing pressure is expected to reduce *U*/*W* and thereby strengthen the coherence of this state,^6^ whereas disorder and possible orbital-selective Mott tendencies, not included explicitly in *H*-, could destabilize it at low temperature and will be interesting topics for future work.

**Figure 7:**
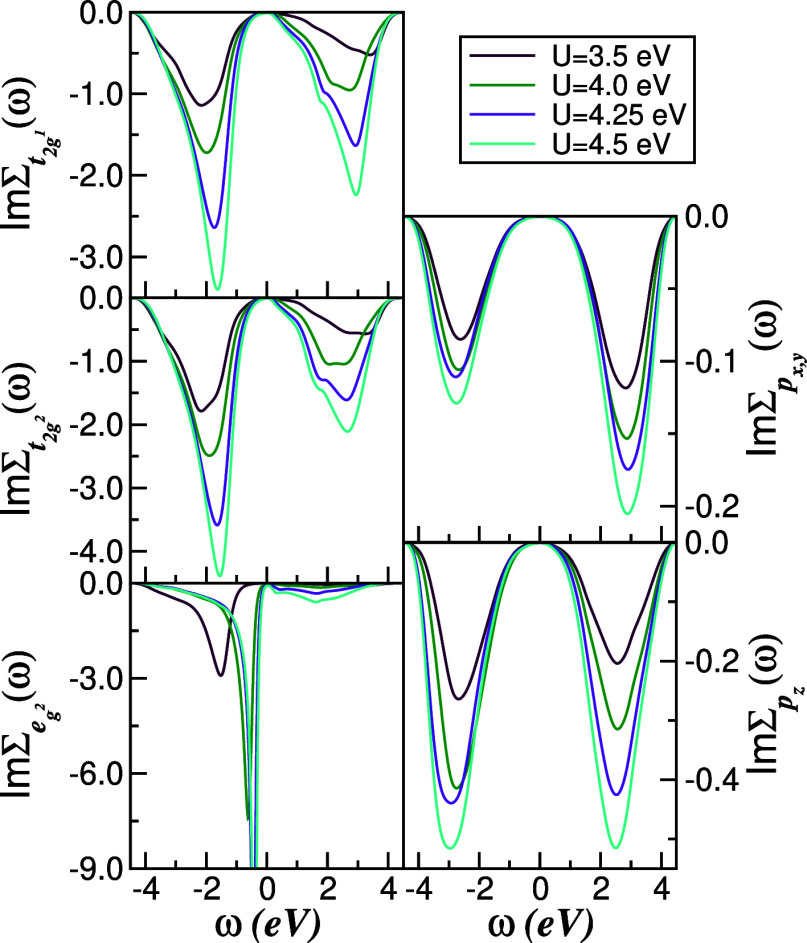
Orbital- and channel-resolved self-energy imaginary parts of 1*T*-TiTe_2_. As in 1*T*-TiSe_2_, Im Σ_α_(ω) follows an ω^2^ law at low frequency, indicative of Landau–Fermi-liquid quasiparticles, despite the reduced *U* values used for the Te system.

To place the correlated electronic structure on a more quantitative footing, Figure 8 compares the DFT and DFT + DMFT total DOS of 1*T*-TiTe_2_ with angle-integrated PES spectra recorded with He II and Al *K*α radiation from ref 8. Over the full valence-band region, the DFT + DMFT curves provide a qualitatively accurate description of the experimental data. In particular, the position of the van Hove-like maximum near −1.6 eV measured with He II photons is well reproduced by the DFT-derived spectral function, while the additional redistribution of intensity captured by DFT + DMFT accounts for a correlation-driven transfer of spectral weight away from this peak. Some discrepancies at the lowest binding energies can be traced back to the simplified treatment of surface and background contributions in the theory, which are typically removed or smoothed in experimental analyses. Such subtleties complicate a strictly quantitative comparison but do not alter the conclusion that the two-band MO model provides a realistic description of the valence electronic structure of 1*T*-TiTe_2_, consistent with more detailed spectroscopic studies in the literature.^61^

**Figure 8:**
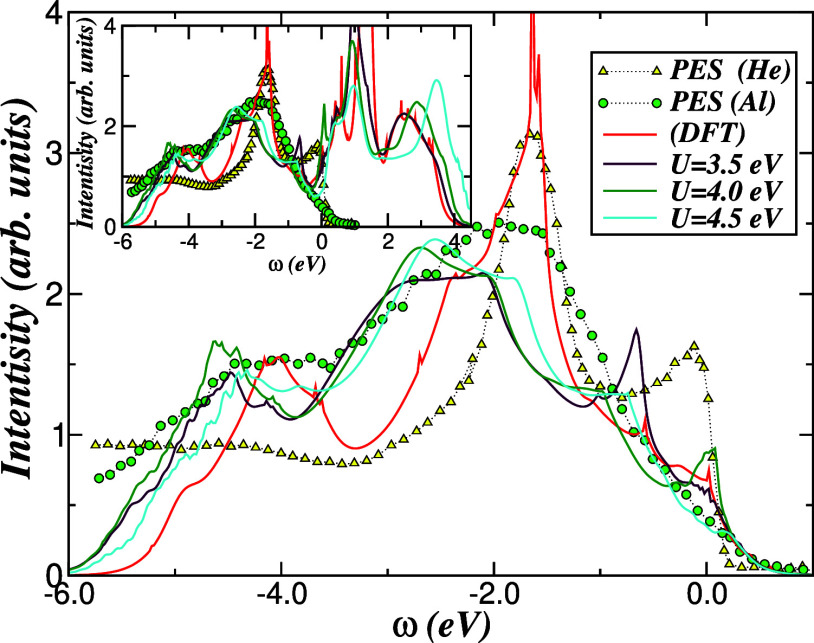
Comparison between the total DFT and DFT + DMFT DOS and angle-integrated photoemission spectroscopy (PES) measured with He II and Al *K*α radiation.^8^ The theory reproduces the overall valence-band line shape of 1*T*-TiTe_2_, including the van Hove peak near −1.6 eV and the correlation-induced spectral-weight transfer. The inset highlights the redistribution of the spectral weight between the valence and conduction bands. PES data adapted with permission from ref 8 (copyright 1996 American Physical Society).

The Landau–Fermi-liquid character of the correlated state is also reflected in the temperature dependence of the *dc* resistivity. Within DMFT,^62^ the static *dc* conductivity can be written in terms of the orbital-resolved spectral functions Aa(k,ω)=−1πImGa(k,ω) as

$$\sigma_{dc}\left(T\right)=\frac{\pi }{T}\underset{a}{\sum }\int d\epsilon \overset{\left(0\right)}{\underset{a}{\rho }}\left(\epsilon \right)\int d\omega \overset{2}{\underset{a}{A}}\left(\epsilon ,\omega \right)f\left(\omega \right)\left[1-f\left(\omega \right)\right]$$

where ρ_*a*_^(0)^(ϵ) is the noninteracting (DFT) DOS of orbital *a* (Figure 3) and *f*(ω) denotes the Fermi function.

Figure 9 displays the resulting *T*-dependent resistivity ρ_*dc*_(*T*) ≡ 1/σ_*dc*_(*T*), normalized to its room-temperature value, for both 1*T*-TiSe_2_ and 1*T*-TiTe_2_. The calculation uses the Ti-3d DFT + DMFT spectral functions and reveals a noticeable *U* dependence below ∼50 K. At intermediate temperatures, the resistivity is roughly linear down to ∼100 K. In 1*T*-TiSe_2_ a clear *T* ^2^ dependence, characteristic of a good LFL metal,^2^ emerges below about 25 K. In contrast, 1*T*-TiTe_2_ exhibits a shallow S-shaped deviation from perfect *T* ^2^ behavior, reminiscent of the response of weakly pseudogapped metals.^63^ Within our DFT + DMFT­(MO-IPT) framework, the calculated ρ_*dc*_(*T*) for 1*T*-TiSe_2_ provides a natural description of the normal-state resistivity above the superconducting transition temperature *T*_c_ in Cu-doped TiSe_2_,^13^ while the emergent S-shape in 1*T*-TiTe_2_ at *U* = 3.5 eV can be viewed as a manifestation of the slightly larger *U*/*W* ratio in the Te compound relative to the Se analogue. Interestingly, experimental measurements of the in-plane resistivity of superconducting TiTe_2_ report deviations from canonical *T* ^2^ scaling below ∼10 K,^12^ in qualitative agreement with our theoretical trends, despite the fact that electron–phonon interactions and the CDW ordered state,^64^ both of which could further modify the low-temperature transport, are not explicitly included in the present modeling.

**Figure 9:**
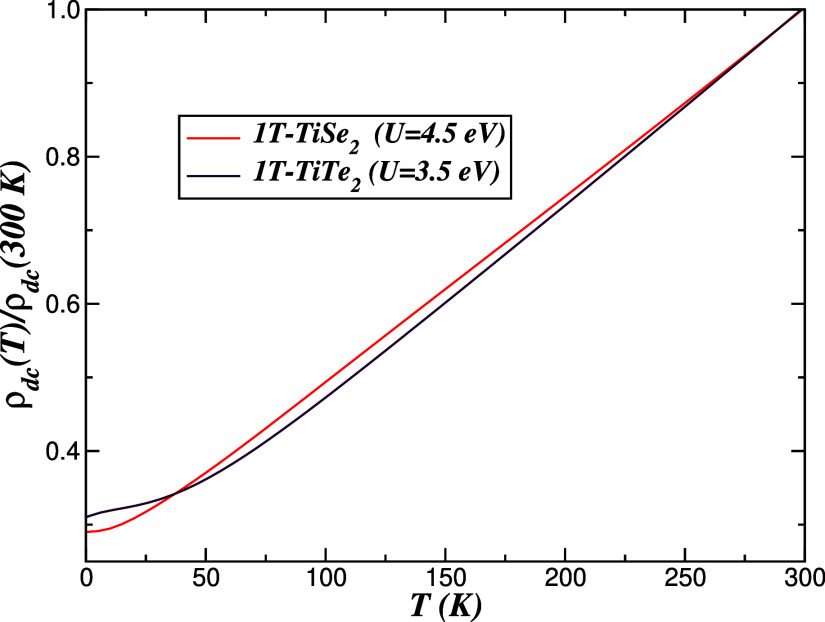
Temperature dependence of the electrical resistivity (normalized to its value at 300 K) for 1*T*-Ti*X*_2_ vdW bulk crystals, computed from the Ti-3d DFT + DMFT spectral functions. For 1*T*-TiSe_2_ an almost perfect *T* ^2^ Landau–Fermi-liquid behavior is obtained below ∼25 K, whereas 1*T*-TiTe_2_ shows a weak S-shaped deviation from *T* ^2^, reflecting its enhanced correlation strength and proximity to a pseudogapped metal.

From a broader perspective, the CDW order that develops in 1*T*-Ti*X*_2_ can be viewed as instability of the correlated LFL state described here. Following the arguments by Sun et al.,^65^ a Fermi liquid with well-defined quasiparticles and Fermi surfaces can undergo instabilities toward nematic, CDW, or stripe-like phases, depending on the dominant ordering channel. In this weak-coupling picture, the CDW phase is an analogue of a stripe phase, sharing the same broken lattice symmetry but emerging from an itinerant parent state with coherent quasiparticles. In the present context, this implies that a direct quantum phase transition from the LFL state identified in our DFT + DMFT analysis to a CDW phase is plausible in 1*T*-TiSe_2_, consistent with earlier model calculations that combined CDW order and correlation-driven band reconstruction.^66^ Exploring the consequences of a coexisting CDW and *s*-wave superconducting phase, as inferred for Cu_*x*_TiSe_2_ in ref 67, is an interesting avenue for future work that goes beyond the scope of the present normal-state study.

## Conclusions

In summary, we have combined DFT and DMFT to investigate the correlated electronic structure of 1*T*-Ti*X*_2_ (*X* = Se, Te) vdW crystals within a realistic multiband, multiorbital framework. Our primary aim has been to elucidate the emergence and nature of the Landau–Fermi liquid state in the normal, non-CDW phase and to clarify how it differs between the Se and Te compounds. The calculations show that local electron–electron interactions in the Ti-3d manifold, supplemented by an intersite Ti–*X* repulsion, drive a pronounced orbital-selective reconstruction of the spectral functions. In 1*T*-TiTe_2_, where the effective ratio *U*/*W* is larger, this leads to a more substantial transfer of spectral weight from low to high energies than in 1*T*-TiSe_2_, and to the formation of channel-selective Hubbard bands in both the valence and conduction sectors. These high-energy features should be accessible to future spectroscopic experiments and provide a direct test of the present modeling.

The coexistence of narrow low-energy resonances with high-energy Hubbard satellites underscores the idea that “more is different”^68^ even in apparently conventional metals: the Te compound, in particular, realizes a multiorbital Fermi liquid whose correlations are strong enough to generate sizable incoherent weight yet still preserve coherent quasiparticles at *E*_F_. Within this LFL scenario, our analysis indicates that retarded interactions associated with collective plasmon excitations^69^ play only a secondary role in determining the low-energy quasiparticle scattering rates,^7^ which are instead dominated by local multiorbital correlations. Targeted studies of collective charge excitations in 1*T*-Ti*X*_2_, for example, via inelastic X-ray or electron scattering,^70^ would be highly valuable to further test this conclusion and refine our understanding of correlated charge dynamics in these vdW metals.

More broadly, the present DFT + DMFT study provides a microscopic starting point for analyzing instabilities of the LFL state toward CDW order and superconductivity in 1*T*-TiSe_2_ and 1*T*-TiTe_2_. Extending the model to explicitly include lattice degrees of freedom and superconducting pairing channels will be essential to connect the normal-state Landau–Fermi-liquidness uncovered here with the rich phase diagrams observed experimentally and to assess the potential of Ti-based dichalcogenides as platforms for correlated-electron devices.

## #

Leibniz Institute for Solid State and Materials Research Dresden, D-01069, Dresden, Germany
